# Hemoptysis and Unilateral Pulmonary Infiltrates due to Severe Acute Mitral Regurgitation from Papillary Muscle Rupture

**DOI:** 10.1155/2024/5534308

**Published:** 2024-01-09

**Authors:** Joanna Wieckowska, Nicholas Diloreto, Shannon Hood, Isabella Chojnacki, Dalia Zakri

**Affiliations:** ^1^Department of Pulmonary/Critical Care, Ascension Genesys Hospital, Grand Blanc, MI, USA; ^2^Department of Graduate Medical Education, Michigan State University School of Osteopathic Medicine, Lansing, MI, USA; ^3^Department of Internal Medicine, Ascension Genesys Hospital, Grand Blanc, MI, USA

## Abstract

Acute mitral regurgitation typically presents with dyspnea, chest pain, and hemodynamic instability. It is an uncommon cause of hemoptysis. We present a case of a patient presenting with dyspnea and hemoptysis without hemodynamic instability along with right-sided infiltrate on chest radiography a few days after an acute inferolateral STEMI who was found to have posterior papillary muscle rupture resulting in acute mitral regurgitation. Our case illustrates that the aforementioned symptoms and signs should raise concern for acute mitral regurgitation and prompt cardiac evaluation in the appropriate clinical setting as they may mimic acute pulmonary processes and delay critical diagnosis and treatment.

## 1. Introduction

Acute mitral regurgitation (AMR) is considered a medical and surgical emergency. Patients often present with hemodynamic instability due to sudden increase of pressure and volume load on the left atrium [1, 2]. Some cases of AMR have presented with sudden onset dyspnea and hemoptysis without hemodynamic instability and shock. AMR is an uncommon cause of hemoptysis, and nontraumatic cases are exceedingly rare [3–6]. AMR has also been seen to present with focal or unilateral pulmonary infiltrates, representing either cardiogenic pulmonary edema or, rarely, diffuse alveolar hemorrhage [3, 7–10]. We present a case of AMR with an atypical presentation and without hemodynamic instability.

## 2. Case Presentation

The patient is a 75-year-old male with a history of thoracic aortic aneurysm, congenital solitary right kidney, lung cancer status post right upper lobe lobectomy (2014), remote prostate cancer status post prostatectomy, hypertension, and coronary artery disease, who presented to the hospital due to sudden onset shortness of breath with fever, cough, and hemoptysis.

Of note, the patient had been recently hospitalized for 4 days as a result of an acute inferolateral ST elevation myocardial infarction (STEMI) requiring drug-eluting stent (DES) to ramus intermedius as well as balloon angioplasty and penumbra thrombectomy of right coronary artery (RCA) complicated by new-onset paroxysmal atrial fibrillation with rapid ventricular response (RVR). He had been subsequently discharged on both dual-antiplatelet therapy (DAPT) and Eliquis.

**Figure 1:**
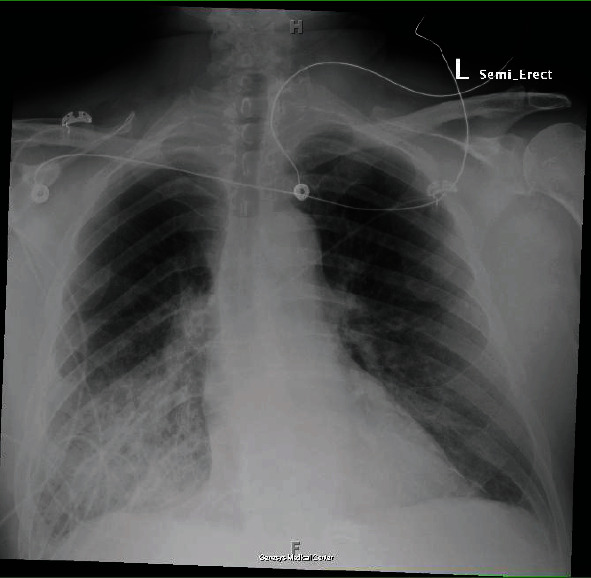
Anteroposterior CXR shows confluent airspace opacities in the right lower lung demonstrating hemorrhage after papillary muscle rupture.

**Figure 2:**
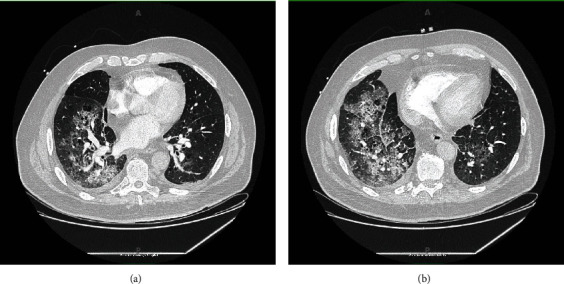
(a, b) CTA chest showing airspace opacities in the right upper lobe and right lower lobe as well as minimally in the left lower lobe, small pleural effusions bilaterally.

**Figure 3:**
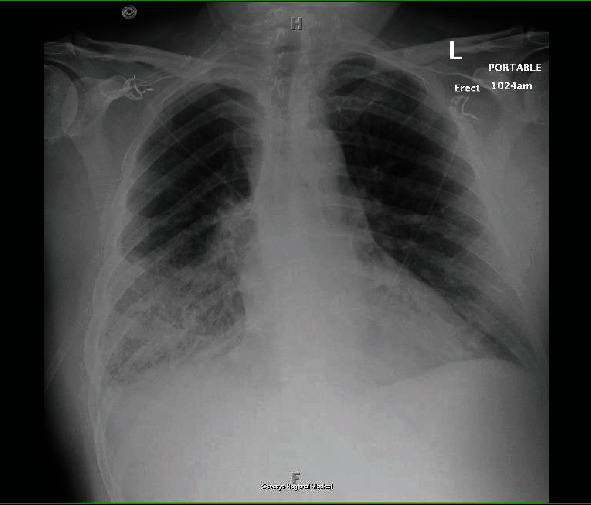
Anteroposterior CXR shows interval worsening of airspace opacities in the right lower lobe.

**Figure 4:**
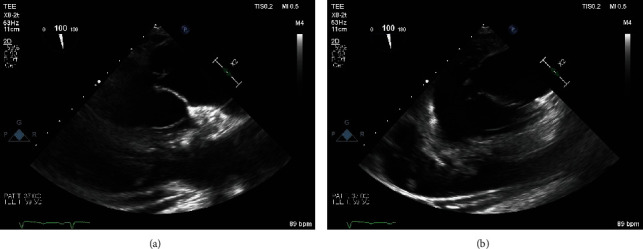
(a, b) TEE showing ruptured head of the posterior papillary muscle prolapsing into the left atrium in systole and back into the left ventricle in diastole.

**Figure 5:**
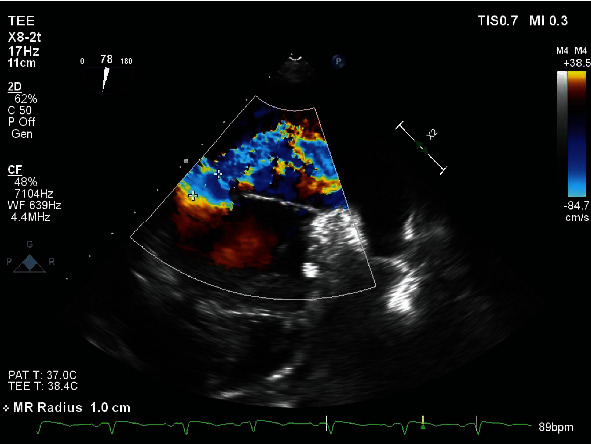
TEE with color Doppler showing severe eccentric mitral regurgitation.

On admission, he was hypoxic, requiring oxygen supplementation using a low-flow nasal cannula, otherwise hemodynamically stable. Labs revealed troponin 11.33 ng/mL (ref: 0-0.04 ng/mL), BNP 516 pg/mL (ref: <100 pg/mL), INR 1.41 (ref: 0.8-1.1), PT 15.9 seconds (ref: 10–13 seconds), LDH 449 IU/L (ref: 140 to 280 IU/L), and WBC 18.6 cells/*μ*L (ref: 4.5-11 cells/*μ*L). Chest radiography (CXR) showed confluent airspace opacities in the right lower lung, concerning for pneumonia, asymmetric pulmonary edema, or pulmonary hemorrhage (Figure 1). CT angiography (CTA) chest revealed airspace opacities in the right upper lobe and right lower lobe as well as minimally in the left lower lobe, small pleural effusions bilaterally, no pulmonary emboli (Figure 2). Transthoracic echocardiogram (TTE) showed ejection fraction (EF) 55-60%, basal inferior wall hypokinesis, and a structurally normal mitral valve with moderate mitral regurgitation. Previous TTE (before percutaneous coronary intervention during previous admission) had shown EF 45-50%, basal inferior and inferolateral wall hypokinesis, and only mild mitral regurgitation. He was started on broad-spectrum antibiotics and diuretics as his acute respiratory failure was concerning for pneumonia and/or cardiogenic pulmonary edema. He was continued on DAPT and Eliquis. However, he began experiencing more frequent epistaxis and hemoptysis. Despite holding anticoagulation, patient began to suffer worsening respiratory distress—tachypnea with respiratory rate in the 30 s, accessory muscle use, and moderate hypoxemia (PaO_2_/FiO_2_ 160 mmHg)—requiring continuous bilevel positive airway pressure (BiPAP) ventilation with an FiO_2_ of 50% in order to alleviate work of breathing and correct hypoxemia. Repeat CXR findings showed interval worsening of airspace opacities in the right lower lobe (Figure 3). He then went into atrial fibrillation with RVR with rates ranging between 120 and 170 s. He was transferred to the ICU, where cardiology performed a transesophageal echocardiogram (TEE) that revealed the head of the posterior papillary muscle to be ruptured and prolapsing into the left atrium in systole and back into the left ventricle in diastole, compatible with severe eccentric mitral regurgitation (Figures 4 and 5). Cardiology subsequently placed an intra-aortic balloon pump (IABP). Shortly after IABP placement, the patient underwent elective intubation in order to undergo mitral valve replacement (MVR) and coronary artery bypass grafting (CABG) ×2.

On postoperative day (POD) 1, the patient was successfully extubated and the IABP was removed. He was transferred out of the ICU on POD 3. Repeat echocardiogram on POD 6 showed left ventricular EF 55-60% with only trace mitral regurgitation. On POD 10, the patient was safely discharged home.

## 3. Discussion

AMR is defined by sudden backflow of blood into the left atrium during systolic contraction causing a reduced forward cardiac output and heart failure. It is a cardiac emergency requiring surgical repair as definitive treatment [1, 2]. Patients with AMR usually present with acute onset of shortness of breath and hypotension. Atypical presentations of AMR have been associated with unilateral lobar consolidation and alveolar hemorrhage [1, 3, 4, 11]. Consistent with the previous literature, our case shows a symptom constellation of sudden dyspnea, hemoptysis, and right-sided infiltration. While a cardiac etiology for hemoptysis is uncommon, an explanation of this symptom may come from the sudden increased pressure from the MR jet into the left atrium and subsequently the pulmonary venous system [11]. Evaluation of AMR with TEE showed an increased elevation pressure gradient directed at the orifice of the right pulmonary venous system compared to the left [5]. This sudden increase in right-sided pulmonary venous pressure can explain the process formation of right-sided pulmonary edema and alveolar hemorrhage [3, 4, 6, 11].

Our case is unique in that the patient presented with atypical symptoms of dyspnea with hemoptysis post inferior STEMI while hemodynamically stable. Initially, our differential diagnoses revolved around a pulmonary source. However, when the patient's respiratory distress worsened despite treatment, we began to suspect another etiology. Although our patient's hemoptysis could have derived from multiple factors, our suspicions of its etiology veered towards cardiac and possible alveolar hemorrhage. Even though our patient had been on DAPT and Eliquis for a few days due to his recent STEMI requiring DES and new diagnosis of atrial fibrillation, we doubted the contribution of these medications contributing to his symptoms as stopping them did not improve his clinical condition and in fact he continued to decline. Furthermore, his history of lung cancer with subsequent right upper lobectomy had happened several years ago and been deemed stable without recurrence, leading us away from suspecting this as a contributing factor to his symptoms.

Surprisingly, the CXR and CTA chest findings (Figures 1–3) were consistent with numerous reports of AMR [3–11]. Initial cardiac evaluation with TTE showed structurally normal mitral valve with moderate mitral regurgitation. However, subsequent TEE confirmed the presence of severe mitral valve regurgitation with papillary rupture, and he underwent definitive treatment with surgical MVR that completely resolved his symptoms. While TTE is the standard of care for initial cardiac workup, it is not as sensitive as TEE and cannot rule out AMR in the setting of papillary muscle rupture [12]. TTE can identify a papillary muscle rupture with a diagnostic sensitivity of 65-85%; TEE is a valuable adjunct as it provides more detailed anatomic information and improves the diagnostic yield to 95–100%, especially if the damage is subtle [12, 13]. Therefore, hemoptysis with right-sided infiltration on CXR and/or CT chest indicative of pulmonary edema or alveolar hemorrhage, even without hemodynamic stability, should raise clinical concern for AMR and prompt cardiac evaluation to prevent the delay of necessary intervention in these critical patients.

## Data Availability

There is no data associated with this article.
